# Prediction of clinical progression of subjective cognitive decline through alterations in morphology and structural covariance networks

**DOI:** 10.1002/brb3.3408

**Published:** 2024-02-05

**Authors:** Zheqi Hu, Xue Zhang, Xinle Hou, Lianlian Wang, Haifeng Chen, Lili Huang, Dan Yang, Yuting Mo, Yun Xu, Feng Bai

**Affiliations:** ^1^ Department of Neurology Nanjing Drum Tower Hospital, Affiliated Hospital of Medical School, Nanjing University Nanjing China; ^2^ Department of Neurology, Nanjing Drum Tower Hospital, State Key Laboratory of Pharmaceutical Biotechnology and Institute of Translational Medicine for Brain Critical Diseases Nanjing University Nanjing China; ^3^ Department of Neurology Nanjing Drum Tower Hospital, Clinical College of Jiangsu University Nanjing China; ^4^ Department of Neurology, Nanjing Drum Tower Hospital, Clinical College of Traditional Chinese and Western Medicine Nanjing University of Chinese Medicine Nanjing China; ^5^ Department of Neurology, Nanjing Drum Tower Hospital Clinical College of Nanjing Medical University Nanjing China; ^6^ Geriatric Medicine Center, Affiliated Taikang Xianlin Drum Tower Hospital Medical School of Nanjing University Nanjing China

**Keywords:** cortical thickness, hippocampal subfields, network‐based statistic, prediction, structural covariance networks, subjective cognitive decline

## Abstract

**Background:**

Subjective cognitive decline (SCD) is a preclinical, asymptomatic stage of Alzheimer's disease (AD). Early identification and assessment of progressive SCD is crucial for preventing the onset of AD.

**Methods:**

The study recruited 60 individuals diagnosed with SCD from the Alzheimer's Disease Neuroimaging Initiative (ADNI) database. Participants were divided into two groups: progressive SCD (pSCD, 23 individuals) and stable SCD (sSCD, 37 individuals) based on their progression to mild cognitive impairment (MCI) within 5 years. Cortical thickness, volumes of the hippocampus subfield, and subcortical regions were analyzed using T1‐weighted images and the FreeSurfer software. Network‐based statistics (NBS) were performed to compare structural covariance networks (SCNs) between the two groups.

**Results:**

Results showed that the pSCD group showed significant atrophy of the hippocampal‐fimbria (*p* = .018) and cortical thinning in the left transverse temporal (cluster size 71.84 mm^2^, cluster‐wise corrected *p* value = .0004) and left middle temporal gyrus (cluster size 45.05 mm^2^, cluster‐wise corrected *p* value = .00639). The combination of these MRI features demonstrated high accuracy (AUC of 0.86, sensitivity of 78.3%, and specificity of 89.3%). NBS analysis revealed that pSCD individuals showed an increase in structural networks within the default mode network (DMN) and a decrease in structural connections between the somatomotor network (Motor) and DMN networks.

**Conclusion:**

Our findings demonstrate that atrophy of the hippocampus and thinning of the cortex may serve as effective biomarkers for early identification of individuals at high risk of cognitive decline. Changes in connectivity within and outside of the DMN may play a crucial role in the pathophysiology of pSCD.

## Graphical abstract

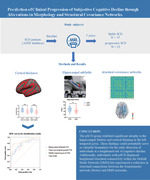

## 1 INTRODUCTION

Alzheimer's disease (AD) is a globally prevalent neurodegenerative disorder that affects over 50 million individuals worldwide (Hodson, 2018). Despite the widespread prevalence of AD, current treatment options remain limited, in part due to the fact that the underlying neuropathological process often begins several years prior to the emergence of cognitive symptoms. Early identification and intervention are critical in the prevention and management of AD, making the preclinical prediction of the disease particularly crucial (Jack et al., 2018; Tondelli et al., 2012). Detection of amnestic mild cognitive impairment (MCI), which is considered to be a preclinical stage of AD, may not be early enough to initiate effective intervention with the fact that significant neuronal loss and irreversible cognitive deterioration may have already occurred at this stage (Ronald C. Petersen, 2009). Subjective cognitive decline (SCD) is defined as an individual's self‐perception of memory and cognitive decline, despite normal results on objective neuropsychological tests. Increasing evidence suggests that the SCD population may be in the early stages of AD and at an increased risk of further cognitive decline and progression to amnestic MCI or AD dementia (Jack et al., 2018; Jessen et al., 2020). A meta‐analysis of a longitudinal SCD study with a follow‐up of at least 4 years showed that the conversion rate of MCI was 27% and that of dementia was 14%, with annual conversion rates of 7% from SCD to MCI and 2% from SCD to dementia (Mitchell et al., 2014). Moreover, cognitively normal individuals with preclinical AD and SCD have a risk of progression to MCI or dementia within three years ranging from 40% to 62% (van Harten et al., 2013; Wolfsgruber et al., 2017). It is a challenging but valuable task to identify individuals with SCD who will progress to MCI or AD (progressive SCD, pSCD) versus those with stable cognitive performance (stable SCD, sSCD) at the baseline.

The focus of research on predicting future cognitive impairment in SCD using biomarkers and neuroimaging markers has been gradually increasing. Despite numerous studies demonstrating the predictive value of cerebrospinal fluid and plasma biomarkers such as β‐amyloid (Aβ) plaques and tau, neurofilament light chain, Aβ positron emission tomography, and tau‐PET in predicting AD progression, their invasive nature, high cost, and limited availability restrict their use to a small number of specialized centers (Dang et al., 2018; Donohue et al., 2017; Karikari et al., 2020; Leuzy et al., 2020; Li et al, 2022; Mattsson‐Carlgren et al., 2020; Palmqvist et al., 2021; Rabinovici et al., 2019).

The existed Aβ and tau in SCD lead to neuronal degeneration (gray matter atrophy), synaptic changes (white matter interruption), and brain functional defects. Based on structural magnetic resonance imaging (sMRI) and functional MRI (fMRI), subtle changes in brain structure and function and the value of classification have been identified, with morphological alterations related to AD (e.g., medial temporal lobe atrophy) detected in SCD subjects (Cherbuin et al, 2015; Dubois et al., 2018; Lim et al., 2019; Shi et al, 2020; Shu et al, 2018; Verfaillie et al., 2016; Yue et al., 2021; Yue et al., 2018). Changes in hippocampal subfields and subcortical regions have also been observed in the trajectory of AD (Zhao et al., 2019; Zhao et al., 2019). Compared with functional data, brain anatomical data are more visible and less susceptible to variations in head movement and the mental state of subjects. Structural covariance network constructed by morphometric parameters (such as gray matter density, volume, and cortical surface extraction) considered to reflect the precise coordination of cortical morphology in the brain and divergent connectivity changes was discovered in AD continuum in recent studies (Evans, 2013; Fu et al., 2021; Kim et al., 2016). However, most studies have utilized cross‐sectional designs rather than longitudinal approaches.

In this longitudinal study, we aimed to investigate the differences between sSCD and pSCD in cortical thickness, hippocampal subfields, and subcortical regions. Furthermore, we will apply network‐based statistic (NBS) to analyze changes in the connectivity of structural covariance networks (SCNs). We hypothesized that alterations in morphometric parameters and SCNs will be observed in pSCD compared to sSCD, thereby favoring the classification of pSCD and sSCD.

## 2 MATERIALS AND METHODS

### 2.1 Participants

The data utilized in this investigation was obtained from the Alzheimer's Disease Neuroimaging Initiative (ADNI) database (ADNI‐2 and ADNI‐3 phase). For more information, see http://www.adni‐info.org. The study was approved by the appropriate Institutional Review Boards at each ADNI site and informed consent was obtained from all participants. This study included 60 individuals with SCD, comprising of 23 pSCDs and 37 sex‐, age‐, and education‐matched sSCDs. The detailed diagnostic criteria can be found in the ADNI manual. In brief, SCD participants reported subjective memory concerns as assessed using the Cognitive Change Index (CCI; total score from the first 12 items ≥ 16), normal cognitive performance on the Mini‐Mental State Examination (MMSE, between 24 and 30), a Clinical Dementia Rating (CDR, score = 0). Regarding memory complaints, normal subjects reported none, whereas both subjects with mild cognitive impairment (MCI) and those with Alzheimer's disease (AD) were required to have complaints. Mini‐Mental State Examination (MMSE) scores ranged from 24 to 30 for normal subjects and subjects with MCI, and from 20 to 26 for AD, with all ranges being inclusive. CDR scores were 0 for normal subjects, 0.5 for subjects with MCI, with a mandatory requirement for the memory box score being 0.5 or greater. The rating for subjects with AD was either 0.5 or 1 (R. C. Petersen et al., 2010). Participants with significant medical, neurological, or psychiatric conditions, such as clinical depression (assessed using the Geriatric Depression Scale‐15 with a score above 5), were excluded from this study (Jack et al., 2018). The progression to MCI or AD within 5 years from baseline was defined an individual as pSCD, while stationary individuals were defined as sSCD.

### 2.2 MRI data acquisition and preprocessing

All MRI scans were acquired using 3.0 Tesla (3.0T) Siemens scanner with subjects were placed in the supine position. A three‐dimensional (3D) T1‐weighted magnetization prepared rapid acquisition gradient echo (MPRAGE) sequence with the following parameters: repetition time (TR) = 8.2 ms, echo time (TE) = 3.0 ms, thickness = 1.0 mm, and number of slices = 176 and no gap, flip angle 9°, 176 slices with no gap, matrix = 256 × 256, voxel size = 1.0 × 1.0 × 1.0 mm^3^.

The structural MRI data underwent surface reconstruction utilizing FreeSurfer 6 (https://surfer.nmr.mgh.harvard.edu/), with the default FreeSurfer command “recon‐all” executed in conjunction with parallel processing commands (Tange, 2011). This processing steps included motion correction, nonlinear spatial normalization to Talairach space, intensity normalization, removal of nonbrain tissue, cortical parcellation, subcortical segmentation, gray and white matter boundary tessellation, automated topology correction, and surface deformation. A comprehensive explanation of the methodology used in this pipeline can be found at https://surfer.nmr.mgh.harvard.edu/fswiki/recon‐all. The cortical thickness was determined by calculating the shortest distance between the gray/white boundary and the gray/cerebrospinal fluid boundary at each vertex on the tessellated surface (Fischl, 2012). Upon discovering a significant difference in cortical thickness between patient groups, the region of interest (ROI) was extracted, mapped onto each subject, and the mean thickness of the ROI was calculated for each individual. Additionally, the cortical thickness for each ROI was extracted using the aparcstats2table routine to construct structural covariance network, utilizing the Schaefer2018_200Parcels_7Networks_order.annot (https://github.com/ThomasYeoLab).

### 2.3 Hippocampal subfields and subcortical regions segmentation

**FIGURE 1:**
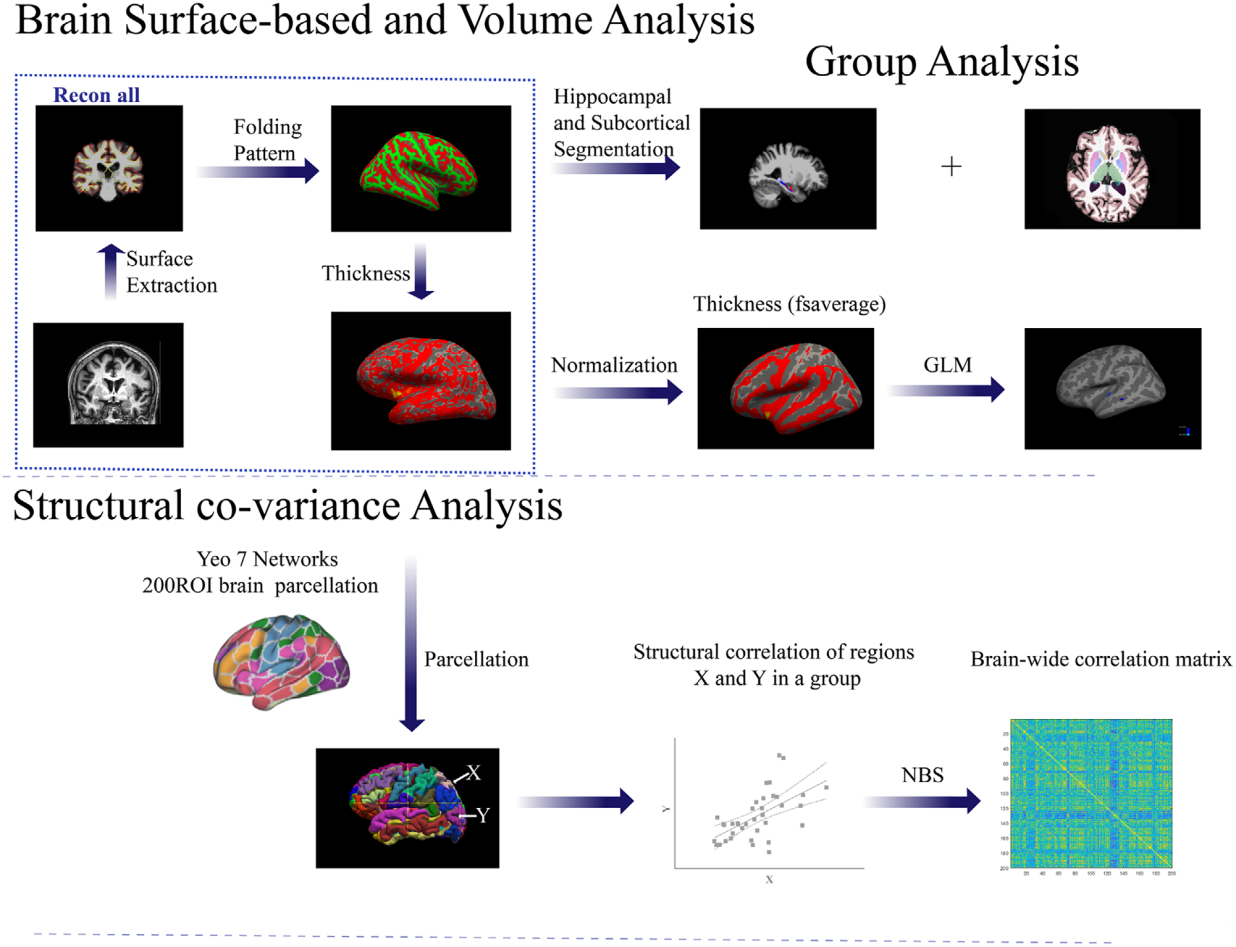
The schematics of the image processing framework and group analysis. GLM, generalized linear model; NBS, network‐based statistic.

Hippocampal subfield segmentation was performed using the integrated hippocampal subfield segmentation software package followed by “recon‐all” (Iglesias et al., 2015). Total of 12 subfields were divided for each side of the hippocampus: the parasubiculum, presubiculum, subiculum, cornus ammonis (CA1, CA3, and CA4) head and body, granule cell layer of the dentate gyrus (GC‐ML‐DG), hippocampus–amygdala transition area (HATA), fimbria, molecular layer, hippocampal‐fissure, and hippocampal tail with quality control performed (Sämann et al., 2022) (Figure 1). Automatic subcortical region segmentation was completed after the default FreeSurfer command “recon‐all,” including Thalamus, Caudate, Putamen, Pallidum, Hippocampus, and Amygdala.

### 2.4 Structural covariance networks construction

A network was established through the combination of nodes and the connections between them. The nodes in the network represented the 200 cortical ROIs that were extracted from the Yeo parcellation in our study (Schaefer et al., 2018). To define the network connections between the nodes, Pearson's correlation coefficients were calculated between the cortical thickness of all possible pairs of ROIs, resulting in a 200 × 200 Pearson's correlation matrix for each group. The information of 200 ROIs was shown in Table S1.

### 2.5 Statistical analysis

#### 2.5.1 Demographic and clinical variables

The measurement data was expressed as the mean ± standard deviation (SD). For the purpose of comparisons between groups, the independent sample *t*‐test and Mann–Whitney *U* test were utilized for normally and nonnormally distributed measurement data, respectively. Meanwhile, the chi‐square (χ^2^) test was used to analyze the differences in gender between groups, which was represented as categorical data. All statistical analyses were performed using SPSS for Windows (version 26.0, IBM), and a significance level of *p* < .05 (two‐tailed) was established.

#### 2.5.2 Hippocampal subfields and subcortical regions

In order to minimize the risk of Type 1 error due to multiple tests, the volume measures, such as the hippocampal subfields and subcortical regions from both hemispheres, were averaged. Furthermore, to control for the impact of individual differences in brain volume on the *t*‐test, each volume was normalized relative to the individual's estimated Total Intracranial Volume (ICV), also referred to as eTIV. This normalization was accomplished by dividing each volume measure by the ICV and expressing it as the normalized volume (*v*), calculated as *v* = (volume raw × 1000) / eTIV (in mm^3^) (Voevodskaya et al., 2014). A significance level of *p* < .05 (two‐tailed) was established for all tests, and the results were corrected for multiple analyses.

#### 2.5.3 Cortical thickness

We utilized the general linear model in FreeSurfer, incorporating a 10‐mm full‐width at half‐maximum Gaussian kernel, to compare cortical thickness between the two groups (pSCD vs. sSCD). A total of 5000 simulations were performed for each comparison for multiple comparisons to define significant clusters (Hagler et al, 2006). Partial correlation analysis was employed (age, gender, and education were the calibration control variables) to see the association between neuropsychological change scores (i.e., MMSE) and MRI features (i.e., thickness and hippocampal subfield of ROI where group difference was found between patient groups).

#### 2.5.4 Network‐based statistic

The NBS approach was employed to examine the existence of a connected subnetwork with altered structural connectivity in pSCD (Zalesky et al, 2010). The primary threshold was initially set to *p* < .005 to identify suprathreshold connections. This process resulted in the identification of all possible connected components in the matrix at an uncorrected level. The statistical significance of the size of each observed component, defined as a set of nodes that can be linked by supra threshold connections, was then determined. The null distribution of maximal component sizes was derived through 10,000 randomized repetitions under the null hypothesis of random group membership. Subnetworks were considered significant at a corrected level of *p* < .05.

#### 2.5.5 Machine learning

The Support Vector Machine (SVM) was selected as the machine learning model for classification, and classification experiments were conducted using the LIBSVM toolbox in MATLAB. The SVM analysis comprised three steps: feature selection, classifier training, and prediction. In our research, statistically significant features such as cortical thickness and volumes of hippocampal subfields were initially chosen for SVM to construct a high‐dimensional space. Subsequently, SVM performed classifier training to establish a hyperplane that optimally separates the classes. Finally, the classifier was employed to predict the class label when a new sample was introduced into the classifier. Due to the limited number of samples, we employed the leave‐one‐out cross‐validation (LOOCV) scheme to assess classifier performance. Assuming there are *N* samples in total, each LOOCV experiment involved excluding one sample, and the remaining *N* – 1 samples were used for model training. Classifier performance was quantified using sensitivity, specificity, and accuracy.

## 3 RESULTS

### 3.1 Demographics and clinical characteristics

The demographic and clinical characteristics of the sSCD and pSCD groups were presented in Table 1. No significant difference was observed in the age (*p* = .339), gender (*p* = .89), education (*p* = .996), CDR scores at baseline or brain tissue volumes (*p* = .596) between the two groups. Furthermore, there were no significant differences in cognitive performance at baseline. At the follow‐up assessment, it was observed that the pSCDs exhibited significantly lower scores on the Montreal Cognitive Assessment (MoCA), CDR, and MMSE compared to the sSCDs (*p* < .001).

**TABLE 1:** Demographics and clinical characteristics.

Items	sSCD (*n* = 37)	pSCD (*n* = 23)	Statistical value	*p* Value
Age (year)	73.16 (5.2)	74.52 (5.55)	−0.964	.339^a^
Gender (male/female)	14/23	10/13	0.23	.890^b^
Education (year)	16.54 (2.51)	16.52 (2.15)	0.03	.976^a^
MMSE (baseline)	29.03 (0.99)	29.3 (1.02)	−1.046	.300^a^
MOCA (baseline)	24.68 (1.68)	23.91 (1.47)	1.786	.079^a^
CDR (baseline)	0	0	–	–
GDS	0.18 (0.94)	1.22 (1.38)	−1.24	.33^c^
CCI	24.35 (7.15)	26 (8.86)	−0.79	.432^a^
eTIV (mm^3^)	1541806.39 (169264.41)	1515934.1 (190715.94)	−0.533	.596^a^
Follow‐up, month	66.32 (19.75)	29.48 (13.77)	7.66	<.001^a^, ^*^
MMSE (follow)	28.86 (1)	27.2 (2.27)	3.57	<.001^a^, ^*^
MOCA (follow)	25.54 (2.65)	22.48 (2.81)	3.43	<.001^a^, ^*^
CDR (follow‐up)	0	1.3	−7.34	<.001^a^

*Note*: Values are presented as the mean ± standard deviation (SD).

^a^ The *p* value was obtained by two‐sample *t*‐test.

^b^The *p* value was obtained by *χ* ^2^ test.

^c^The *p* value was obtained by Mann–Whitney *U* test.

*A statistical difference between groups, *p* < .05.

Abbreviations: CCI, Cognitive Change Index; CDR, Clinical Dementia Rating; eTIV, estimated Total Intracranial Volume; GDS, Geriatric Depression Scale; MMSE, Mini‐Mental State Examination; MoCA, Montreal Cognitive Assessment; pSCD, progressive subjective cognitive decline; sSCD, stable subjective cognitive decline.

### 3.2 Comparison of hippocampal subfield and subcortical regions

**FIGURE 2:**
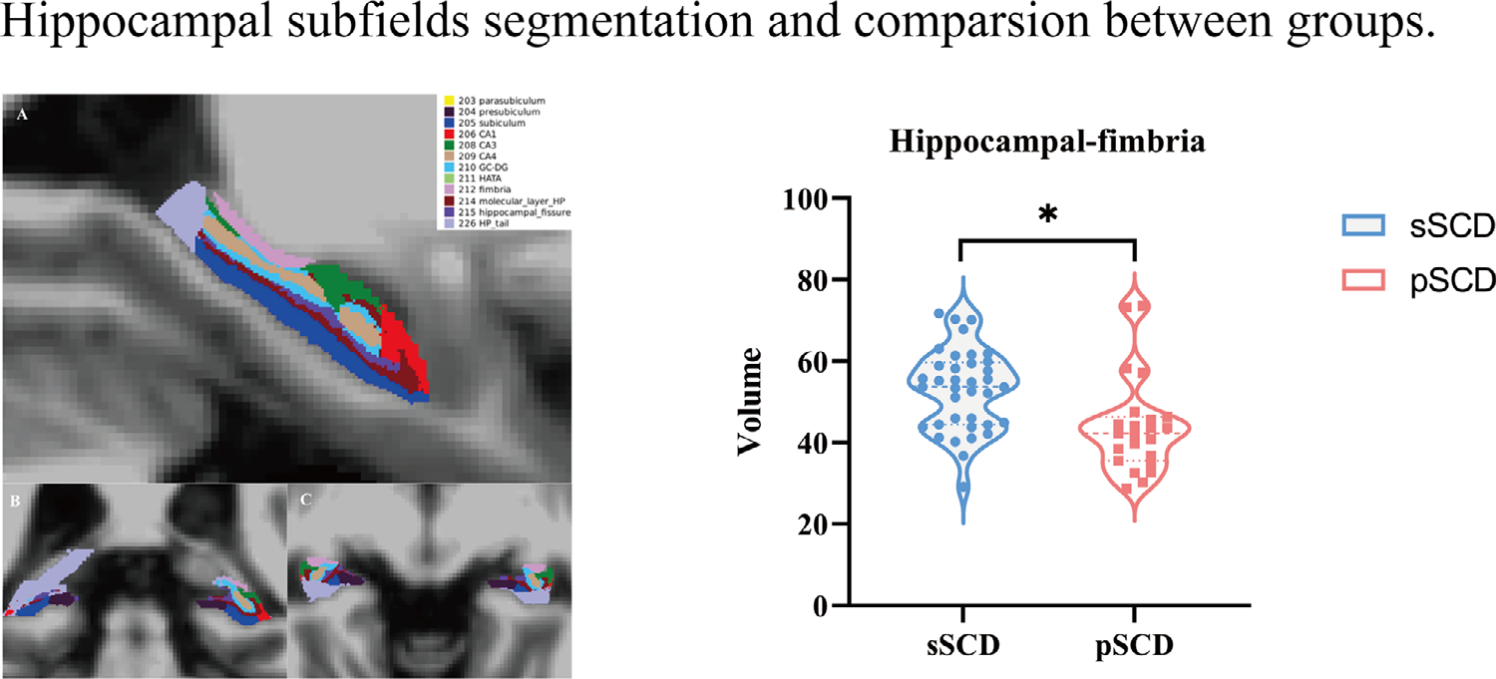
Representative example of hippocampal subfield segmentations from a patient with SCD in different view and decreased volumes in the hippocampal‐fimbria (*p* = .018, FDR corrected) areas. sSCD, stable subjective cognitive decline; pSCD, progressive subjective cognitive decline. *<.05.

We investigated the differences in volumes of the hippocampal subfields and subcortical regions between pSCD and sSCD groups. As depicted (Table 2, Figure 2), compared with sSCD group, the pSCD group showed decreased volumes in the hippocampal‐fimbria (*p* = .018, FDR corrected). A trend toward significant volume reduction between the two groups was observed; however, no statistically significant difference was found in other hippocampal subfields or subcortical regions between the pSCD and sSCD groups.

**TABLE 2:** Comparison of hippocampal subfields and subcortical structure between groups.

Items	sSCD (*n* = 37)	pSCD (*n* = 23)	Statistical value	*p* Value	Adjusted *p* value
Hippocampal_tail	332.96 (49.83)	306.43 (48.47)	2.026	.047	.284^a^
Subiculum	271.71 (33.03)	261.31 (36.6)	1.138	.260	.468^a^
CA1	412.25 (55.85)	386.76 (43.42)	1.865	.067	.284^a^
Hippocampal‐fissure	113.07 (21.01)	113.68 (16.39)	−0.119	.906	.959^a^
Presubiculum	191.28 (23.59)	186.38 (28.66)	0.720	.474	.711^a^
Parasubiculum	42.05 (6.6)	41.73 (7.37)	0.177	.860	.959^a^
Molecular_layer_HP	356.92 (44.35)	338.98 (39.57)	1.630	.164	.352^b^
GC‐ML‐DG	187.03 (24)	176.11 (21.19)	1.790	.079	.284^a^
CA3	135.07 (21.53)	127.61 (18.74)	1.369	.176	.352^a^
CA4	161.65 (20.67)	153.86 (17.7)	1.497	.140	.352^a^
Hippocampal‐fimbria	52.94 (9.95)	43.99 (11.84)	3.022	.001	.018^b^, ^*^
HATA	40.42 (6.56)	38.93 (5.28)	0.917	.363	.594^a^
Whole_hippocampus	2184.29 (254.75)	2062.08 (228.79)	1.877	.066	.284^a^
Thalamus‐proper	4121.78 (468.41)	4124.56 (259.93)	−0.026	.979	.979^a^
Caudate	2174.6 (307.94)	2136.15 (275.64)	0.502	.548	.758^b^
Putamen	2801.01 (399.31)	2774.06 (267.25)	0.286	.776	.959^a^
Pallidum	1239.19 (163.33)	1250.02 (163.42)	−0.250	.804	.959^a^
Amygdala	1074.87 (144.53)	1021.73 (107.22)	1.520	.134	.352^a^

*Note*: Comparison of hippocampal subfields and subcortical structure between sSCD and pSCD group (FDR corrected). In the hippocampal subfields, compared with sSCD group, the pSCD group showed decreased volumes in the hippocampal‐fimbria (*q* = .018, FDR corrected) areas. Values are presented as the mean ± standard deviation (SD).

^a^The *p* value was obtained by two‐sample *t*‐tests.

^b^The *p* value was obtained by Mann–Whitney *U* test.

*A statistical difference between groups, *p* < .05. Normalized volume: *ṽ* = *v* × 1000/eTIV.

Abbreviations: sSCD, stable subjective cognitive decline; pSCD, progressive subjective cognitive decline; GC‐ML‐DG, Molecular and Granule Cell Layers of the Dentate; HATA, Hippocampal‐Amygdaloid Transition Area; eTIV, estimated Total Intracranial Volume.

### 3.3 Comparison of cortical thickness

**FIGURE 3:**
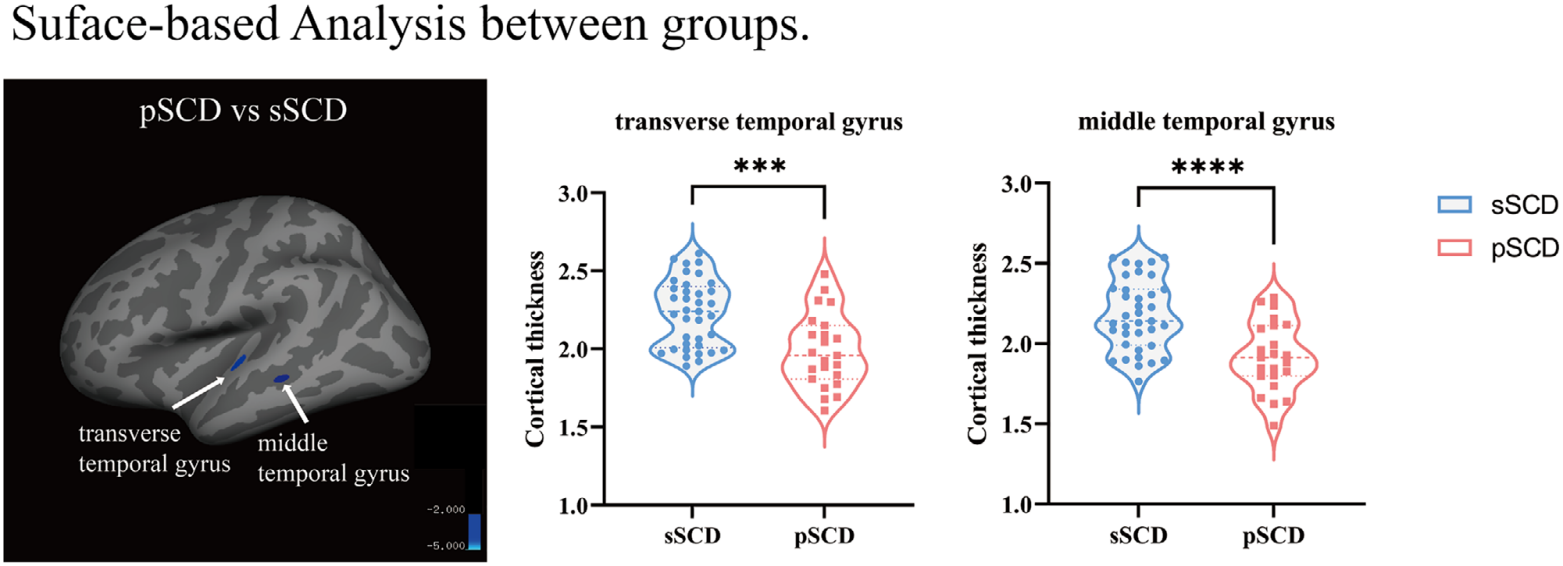
The location of brain regions that differed significantly between groups. sSCD, stable subjective cognitive decline; pSCD, progressive subjective cognitive decline. ***<.001, ****<.0001.

Using general linear model and multiple comparisons, results revealed that the pSCD patients exhibited cortical thinning predominantly in left transverse temporal gyrus (cluster size 71.84 mm^2^, cluster‐wise corrected *p* value = .0004) and left middle temporal gyrus (cluster size 45.05 mm^2^, cluster‐wise corrected *p* value = .00639,) compared with sSCD subjects (Table 3, Figure 3).

**TABLE 3:** Clusters of significant difference in cortical thickness between groups.

Cluster	Max	Size (mm^2^)	MNIX	MNIY	MNIZ	*p*
Left transverse temporal gyrus	−3.781	71.84	−51.2	−15.9	4	.0004
Left middle temporal gyrus	−4.442	45.05	−51.5	−35	−9.3	.00639

### 3.4 Correlation between MRI features and clinical measures

The results of the correlation analysis between cortical thickness and neuropsychological test change scores revealed that no significant correlation was observed. Instead, a tread of negative correlation between hippocampal‐fimbria and MMSE decline was found (*R* = −0.433, *p* = .057, Table 4), which indicated that the more severe the atrophy of hippocampal fields, the poorer the subsequent cognitive levels. Besides, the rate of cognitive decline (measured by cognitive decline values over follow‐up years) was also been incorporated into the statistical analysis, but no statistically significant correlations were found.

**TABLE 4:** Results of discrimination analyses.

Feature	AUC	SEN	SPE	Cut‐off
Hippocampal‐fimbria	0.747	0.6087	0.8378	43.77
Transverse temporal gyrus	0.776	0.8261	0.6216	2.18
Middle temporal gyrus	0.788	0.6957	0.7568	1.994
Fimbria+ transverse+ middle temporal	0.86	0.7826	0.8919	0.465

Abbreviations: AUC, area under the curve; SEN, sensitivity; SPE, specificity.

### 3.5 Value of MRI features to classification

**FIGURE 4:**
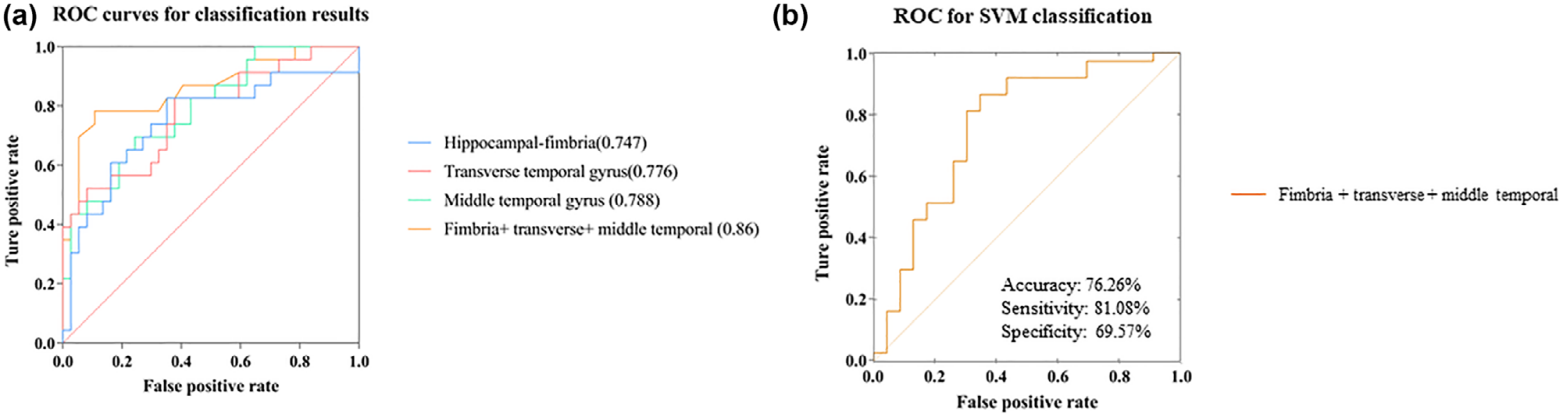
(a) Receiver operating characteristic (ROC) of classification based on different morphological features. The blue curve represents the ROC curve of hippocampal‐fimbria. The red curve represents the ROC curve of transverse temporal gyrus. The green curve represents the ROC curve of middle temporal gyrus. The yellow curve represents the ROC curve of Integration of the above indicators. (b) ROC for SVM classification.

In this article, we tested the classification value of MRI features (Figure 4a and Table 4). For single feature analysis, classification performance was observed in hippocampal‐fimbria (AUC = 0.747, sensitivity = 60.9%, specificity = 83.8%), transverse temporal gyrus (AUC =  0.776, sensitivity = 82.6%, specificity = 62.2%), and middle temporal gyrus (AUC = 0.788, sensitivity = 69.6%, specificity = 75.7%). The accuracy of the classifier was improved after combining the features, with the highest AUC of 0.86, sensitivity of 78.3%, and specificity of 89.3% (Figure 4, Table 4). Based on the image features mentioned above, the SVM model could distinguish the pSCD patients from sSCD with an accuracy of 76.26%, a sensitivity of 81.08%, and a specificity of 69.57% (Figure 4b).

### 3.6 Altered structural covariance network connectivity in pSCD

**FIGURE 5:**
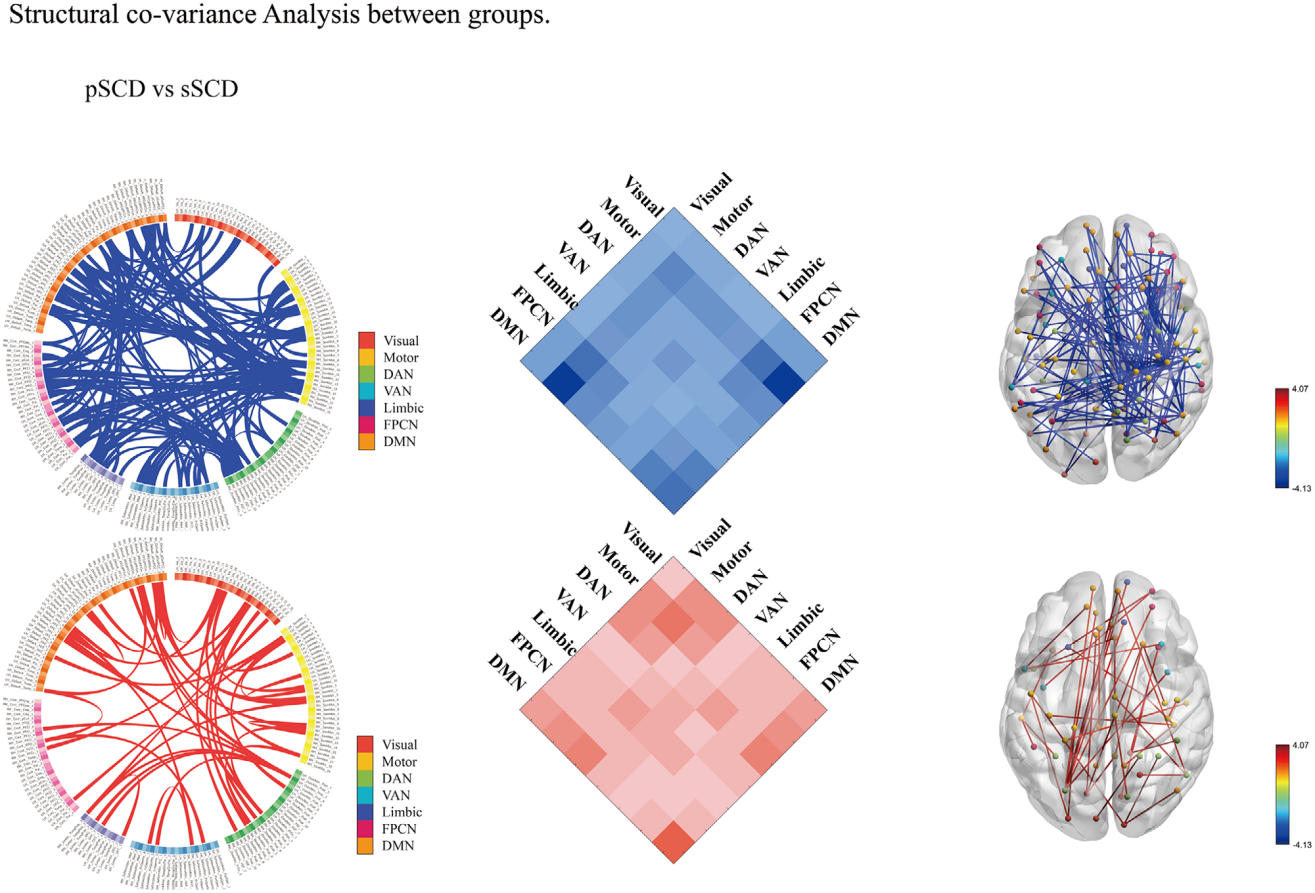
The altered connected subnetwork in patients with pSCD versus cSCD based on the NBS analysis. pSCD patients showed significantly increased connections within the first subnetwork consisting of 103 nodes and 239 edges (*p* = .0314, corrected). The second subnetwork was composed of 54 nodes and 88 edges, which was significantly increased in pSCD compared with the cSCD group (*p* = .0314, corrected). ALS, amyotrophic lateral sclerosis; HC, healthy controls; NBS, network‐based statistic; Visual, visual network; Motor, somatomotor network; DAN, dorsal attention network; VAN, ventral attention network; Limbic, limbic network; VN, visual network; FPCN, frontoparietal network; DMN, default mode network. *Note*: blue lines represent lower connections in SCD than HCs, red lines represent higher connections in SCD than HC.

**FIGURE 6:**
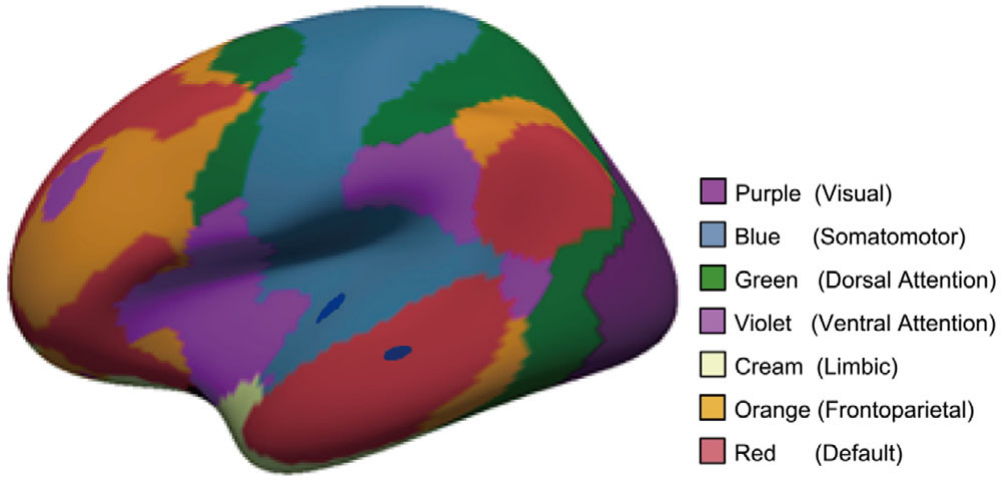
The location of brain regions in the network with significantly different cortical thickness.

The results of the nonparametric NBS analysis revealed significant differences in the SCNs between pSCD and sSCD groups (Figure 5). The pSCD group showed significant decreases in connections within the first subnetwork (*p* = .0314, corrected), which comprised 103 nodes and 239 edges, and was predominantly composed of the interregion connections between the Motor network and the DMN (edges: 32/239, 13.39%). It is noteworthy that the transverse temporal gyrus is situated within the Motor network, whereas the middle temporal gyrus is positioned within the DMN (Figure 6). The second subnetwork, composed of 54 nodes and 88 edges, was significantly increased in the pSCD group (*p* = .0314, corrected) and was mainly involved within the DMN (edges: 8/88, 9.09%).

## 4 DISCUSSION

In this study, we aimed to investigate the differences between subjects with sSCD and pSCD using sMRI and to examine their value for classification. Our findings revealed that (i) compared to sSCD, pSCD individuals exhibited decreased volumes in the hippocampal‐fimbria and cortical thinning in the left temporal lobe (specifically, the transverse and middle temporal gyrus). The integration of these differences led to improved accuracy in predicting pSCD. (ii) Results from NBS analysis showed that compared to sSCD, pSCD individuals had significantly increased structural connections predominantly within the default mode network and decreased connections primarily between the motor network and DMN. These findings have implications for early identification and individualized interventions for those at high risk of Alzheimer's disease.

### 4.1 Volumetric comparisons of hippocampal subfields and subcortical regions

Previous investigations have reported that subfields within the hippocampus demonstrate a trend toward significant volume reduction in the progression of AD (Zhao et al., 2019). Atrophy of CA1, subiculum, and presubiculum represented the earliest sites for AD pathologic changes (i.e., amyloid deposits, tau aggregation) ( Braak & Del Tredici, 2020; Furcila et al, 2019; Izzo et al, 2020). In this study, we assessed the volumetric differences between subfields within the hippocampus and subcortical regions between two groups. Results showed that the pSCD group exhibited a decrease in volume in the fimbria, a small bundle of white matter fibers along the superior surface of the hippocampus. The fimbria serves as a structural connection between the hippocampus and the rest of the brain and its integrity is important in preserving the hippocampus’ role in memory (Nilsson et al, 1987; Tatu & Vuillier, 2014). Although a trend toward volume reduction was observed in the pSCD group, there were no statistically significant differences in other subfields within the hippocampus or subcortical regions after multiple comparisons. Further analysis of the changes in subfields within the hippocampus and subcortical regions from baseline to MCI stage in the pSCD group revealed that the CA1, CA3, CA4, GC‐ML‐DG, HATA, molecular_layer_HP, subiculum, hippocampus, and amygdala were significantly larger at baseline compared to MCI stage (Figure S1). These findings are consistent with previous studies (Yue et al., 2018; Zhao et al., 2019). It is suspected that the shrinkage of the hippocampal subregions is not synchronous, with the alteration of the fimbria in the early stages being one possible explanation for the shrinkage of other subregions.

### 4.2 Comparison of cortical thickness

The onset of AD is typically characterized by changes in brain structure that may occur years or decades prior to the clinical manifestation of the disease. A specific pattern of cortical thinning, referred to as the “cortical AD‐signature,” has been identified as a potential early marker of the disease and has been shown to be associated with an increased risk of clinical progression in patients with SCD(Bakkour et al, 2009; Dickerson et al., 2011). In general, this proposed AD‐signature was associated with an increased risk of clinical progression in SCD patients. Cross‐sectional studies have demonstrated that patients experiencing SCD exhibited a reduction in cerebral blood flow and an increase in functional connectivity in the left middle temporal gyrus compared to the health control group (W. Li et al., 2022; S. Wang et al., 2021). Additionally, another investigation revealed that individuals with multidomain SCD demonstrated elevated regional amyloid SUVr in the left middle temporal gyrus in comparison to those with single memory domain SCD (X. Wang et al., 2022). However, it remains unclear whether changes in cortical thickness occur in patients with pSCD at baseline compared with sSCD group. In this study, we found significant thinning in the left transverse and middle temporal gyrus in participants with pSCD. This suggests that cortical thinning begins in the temporal region and is more sensitive to early AD‐related changes compared to other brain regions. The cortical thickness of the temporal region may be useful for the early identification of individuals with pSCD.

We further investigated the predictive value of consensus features for trends in cognitive changes. Cognitive scores during follow‐up may be influenced by the baseline cognitive levels; therefore, we calculated the Pearson's correlation coefficient between consensus features and neuropsychological test change scores. Besides, the rate of cognitive decline (measured by cognitive decline values over follow‐up years) was also been incorporated into the statistical analysis. Results showed that no significant correlation was observed. Instead, a tread of negative correlation between hippocampal‐fimbria and MMSE decline was found, which indicated that the more severe the atrophy of hippocampal fields, the poorer the subsequent cognitive levels. Existing literature suggests that hippocampal volume can predict clinical progression in individuals with subjective cognitive decline (SCD) (Ebenau et al., 2022). Our study, however, reveals that specific subfields of the hippocampus also hold predictive value, albeit without reaching statistical significance. Nevertheless, these findings carry meaningful clinical implications.

### 4.3 Value of MRI features to classification

In this study, we tested the classification value using features, suggesting that individual‐level morphological analysis could hold diagnostic potential for differentiating pSCD from subjects sSCD individuals. The integration of multiple MRI features (cortical thickness and hippocampal‐fimbria) resulted in an improved AUC of 0.86, indicating that combining multiple morphological features could provide complementary information from different brain structures, suggesting potential for clinical utility. Imaging features combined with ML obtained a great classification performance with accuracy of 76.26%. Studies focus on identification subjective cognitive decline from other stage of AD continuum or health individual using simple or multimodal MRI yields excellent and powerful performances (Chen et al., 2022; Lin et al., 2022; Palmqvist et al., 2021). The potential for clinical utility of morphometric features (cortical thickness, subcortical regions) obtained from sMRI in distinguishing pSCD and sSCD was recognized (Yue et al., 2021). In our study, we further found the value of temporal thickness in prediction of pSCD by GLM method. Merging the altered characteristics of the hippocampus subfields our study showed higher predictive value.

### 4.4 Disrupted subnetworks detected by NBS

NBS analysis was used to explore the alterations in structural connections and results revealed that the significantly increased and decreased internodal connections within two subnetworks. There was a subnetwork with decreased connections primarily located between the DMN and the Motor network, while increased connections were observed within the DMN. The DMN, which is involved in memory and self‐related processing, is considered one of the most vulnerable regions in AD (Raichle, 2015). Previous studies have shown that the functional connectivity in the DMN undergoes a nonlinear change trajectory over the progression from subjective to objective cognitive decline in SCD individuals (Viviano & Damoiseaux, 2020). Furthermore, Li et al. (2019) found that along the AD progression, DMN showed increased structural association at the early stage while decreased structural association at the late stage. Early synaptic disruption may lead to compensatory recruitment that underlie increased functional and structural connectivity in AD‐spectrum patients (Sheng et al., 2021; Viviano & Damoiseaux, 2020). Notably, the transverse temporal gyrus exhibits a localized variation within the Motor network, while the middle temporal gyrus manifests a distinct cortical thickness variation within the DMN. We speculate that higher structural connections within the DMN may represent a compensatory mechanism of the decreased connections involved between DMN and Motor network to maintain stable cognitive ability in SCD individuals.

### 4.5 Limitations

The present study has several limitations that need to be considered. First, the sample size of the study is limited, and the findings need to be confirmed with a larger sample size in future studies. Second, we did not collect FDG‐PET or Aβ marker data, which makes it uncertain whether the observed progression was related to AD or not. Finally, this study only included sMRI data, and incorporating multimodal imaging techniques such as fMRI and PET in future studies would provide a more comprehensive understanding of the results.

## 5 CONCLUSION

Our findings showed that individuals with pSCD exhibit significant atrophy in the cortical thickness of the temporal gyrus, as well as decreased volumes in the hippocampus subfields. By combining significant MRI features, we observed a high accuracy in identifying individuals at high risk of cognitive decline, providing potential as effective biomarkers for early stage identification. Furthermore, our results suggest that changes in connectivity both within and outside of the DMN may contribute to morphological changes in these regions.

## AUTHOR CONTRIBUTIONS

**Zheqi Hu**: Conceptualization; methodology; writing—original draft; visualization; supervision; formal analysis. **Xue Zhang**: Conceptualization; methodology; investigation; validation. **Xinle Hou**: Validation; formal analysis; investigation; data curation. **Lianlian Wang**: Software; data curation. **Haifeng Chen**: Visualization; methodology. **Lili Huang**: Methodology. **Dan Yang**: Methodology. **Yuting Mo**: Methodology. **Yun Xu**: Supervision; writing—review and editing; project administration; investigation. **Feng Bai**: Writing—review and editing; resources; funding acquisition; supervision.

## FUNDING

Data collection and sharing for this project was in part funded by the Alzheimer's Disease Neuroimaging Initiative (ADNI) (National Institutes of Health Grant U01 AG024904) and DOD ADNI (Department of Defense award number W81XWH‐12‐2‐0012). ADNI is funded by the National Institute on Aging, the National Institute of Biomedical Imaging and Bioengineering, and through generous contributions from the following: AbbVie, Alzheimer's Association; Alzheimer's Drug Discovery Foundation; Araclon Biotech; BioClinica, Inc.; Biogen; BristolMyers Squibb Company; CereSpir, Inc.; Cogstate; Eisai Inc.; Elan Pharmaceuticals, Inc.; Eli Lilly and Company; EuroImmun; F. Hoffmann‐La Roche Ltd and its affiliated company Genentech, Inc.; Fujirebio; GE Healthcare; IXICO Ltd.; Janssen Alzheimer Immunotherapy Research & Development, LLC.; 1510 W. Yao et al. / Core‐Centered Connection Abnormalities Associated with Pathological Features Mediate Johnson & Johnson Pharmaceutical Research & Development LLC.; Lumosity; Lundbeck; Merck & Co., Inc.; Meso Scale Diagnostics, LLC.; NeuroRx Research; Neurotrack Technologies; Novartis Pharmaceuticals Corporation; Pfizer Inc.; Piramal Imaging; Servier; Takeda Pharmaceutical Company; and Transition Therapeutics. The Canadian Institutes of Health Research is providing funds to support ADNI clinical sites in Canada. Private sector contributions are facilitated by the Foundation for the National Institutes of Health (http://www.fnih.org). The grantee organization is the Northern California Institute for Research and Education and the study is coordinated by the Alzheimer's Therapeutic Research Institute at the University of Southern California. ADNI data are disseminated by the Laboratory for Neuro Imaging at the University of Southern California.

### PEER REVIEW

The peer review history for this article is available at https://publons.com/publon/10.1002/brb3.3408.

## Supporting information

Supplementary Material.

Figure S1. Changes of hippocampal subfields and subcortical regions from pSCD to MCI. Results showed that among the hippocampal subfields and subcortical regions, the CA1, CA3, CA4, GC‐ML‐DG, HATA, molecular_layer_HP, subiculum, hippocampus, and amygdala were significantly larger when pSCD at baseline than MCI stage. *<.05, **<.01.

Supplementary Material.

## Data Availability

The datasets used and/or analyzed during this study are available from the corresponding authors on reasonable request.
