# Supplementary material for: Prediction of clinical progression of subjective cognitive decline through alterations in morphology and structural covariance networks
Source: Brain Behav. 2024 Feb 5;14(2):e3408. doi: 10.1002/brb3.3408 (PMC10839539; doi:10.1002/brb3.3408)
Supplement: Supplementary file 2 — Figure S1. Changes of hippocampal subfields and subcortical regions from pSCD to MCI. Results showed that among the hippocampal subfields and subcortical regions, the CA1, CA3, CA4, GC‐ML‐DG, HATA, molecular_layer_HP, subiculum, hippocampus, and amygdala were significantly larger when pSCD at baseline than MCI stage. *<.05, **<.01. [file BRB3-14-e3408-s003.docx]

**Prediction of Clinical Progression of Subjective Cognitive Decline through Alterations in Morphology and Structural Covariance Networks**

Zheqi Hu^1,2a^, Lianlian Wang^4a^, Xue Zhang^1,2^, Haifeng Chen^1,2,5^, Lili Huang^1,2^, Dan Yang^6^, Yuting Mo^6^, Yun Xu^1,2^, Feng Bai^1,2,3*^

^1^Department of Neurology, Nanjing Drum Tower Hospital, Affiliated Hospital of Medical School, Nanjing University, Nanjing 210008, China.

^2^Department of Neurology, Nanjing Drum Tower Hospital, State Key Laboratory of Pharmaceutical Biotechnology and Institute of Translational Medicine for Brain Critical Diseases, Nanjing University, Nanjing 210008, China.

^3^Geriatric Medicine Center, Affiliated Taikang Xianlin Drum Tower Hospital, Medical School of Nanjing University, Nanjing 210008, China.

^4^Department of Neurology, Nanjing Drum Tower Hospital Clinical College of Jiangsu University, Nanjing, China.

^5^Nanjing Drum Tower Hospital Clinical College of Traditional Chinese and Western Medicine, Nanjing University of Chinese Medicine, Nanjing, China.

^6^Nanjing Drum Tower Hospital Clinical College of Nanjing Medical University, Nanjing, China.

^a^These authors contributed equally to this work,

*Corresponding author: Feng Bai,

E-mail address: baifeng515@126.com.

Address: 321 Zhongshan Road, Nanjing, 210008, Jiangsu Province, China.

Telephone number: 0086-25-83105960.


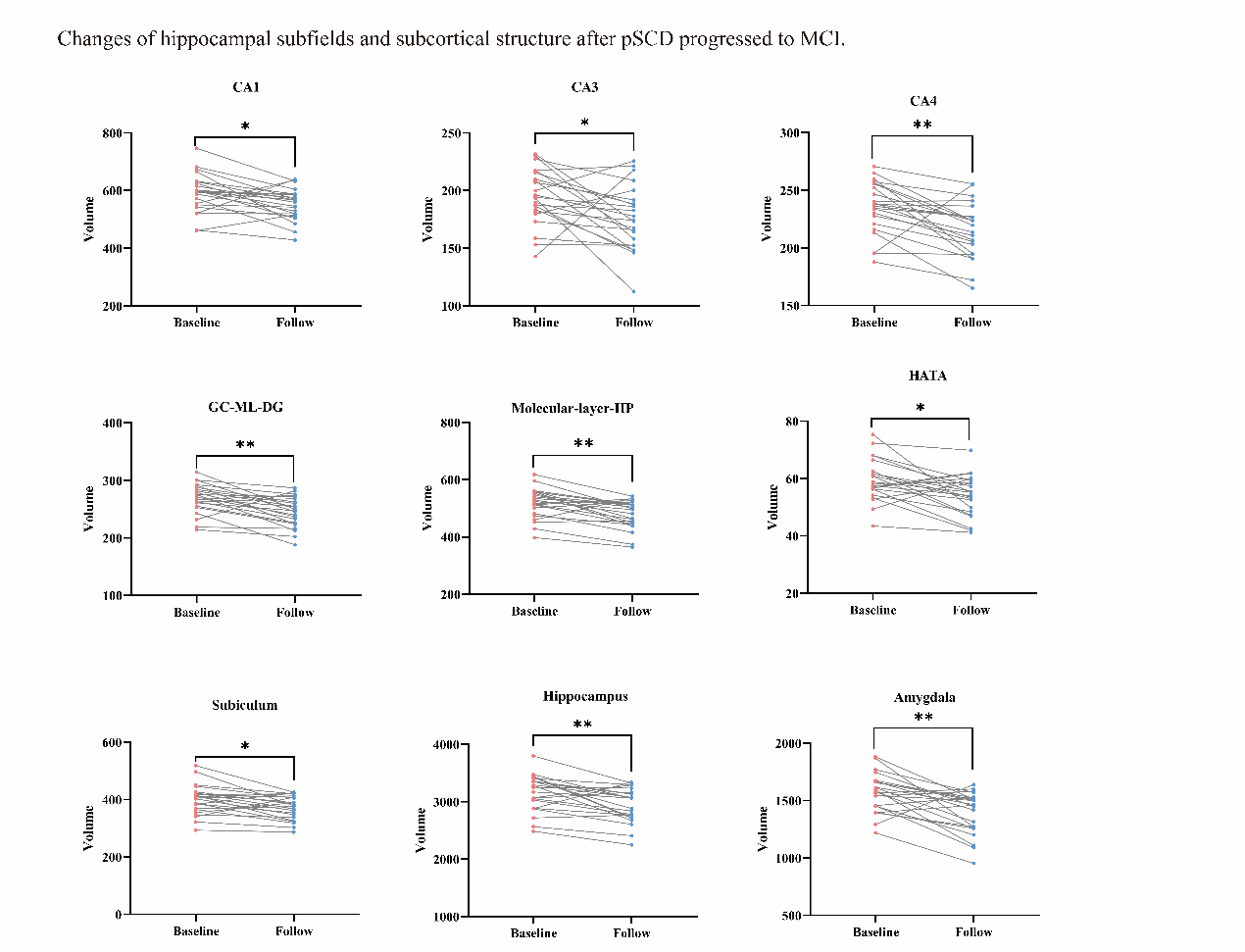


Supplementary Figure1. Changes of hippocampal subfields and subcortical regions from pSCD to MCI. Results showed that among the hippocampal subfields and subcortical regions, the CA1, CA3, CA4, GC-ML-DG, HATA, molecular_layer_HP, subiculum, hippocampus and amygdala was significantly larger when pSCD at baseline than MCI stage. * < 0.05, **<0.01.
