# Supplementary figures and images for: Prediction of clinical progression of subjective cognitive decline through alterations in morphology and structural covariance networks
Source: Brain Behav. 2024 Feb 5;14(2):e3408. doi: 10.1002/brb3.3408 (PMC10839539; doi:10.1002/brb3.3408)

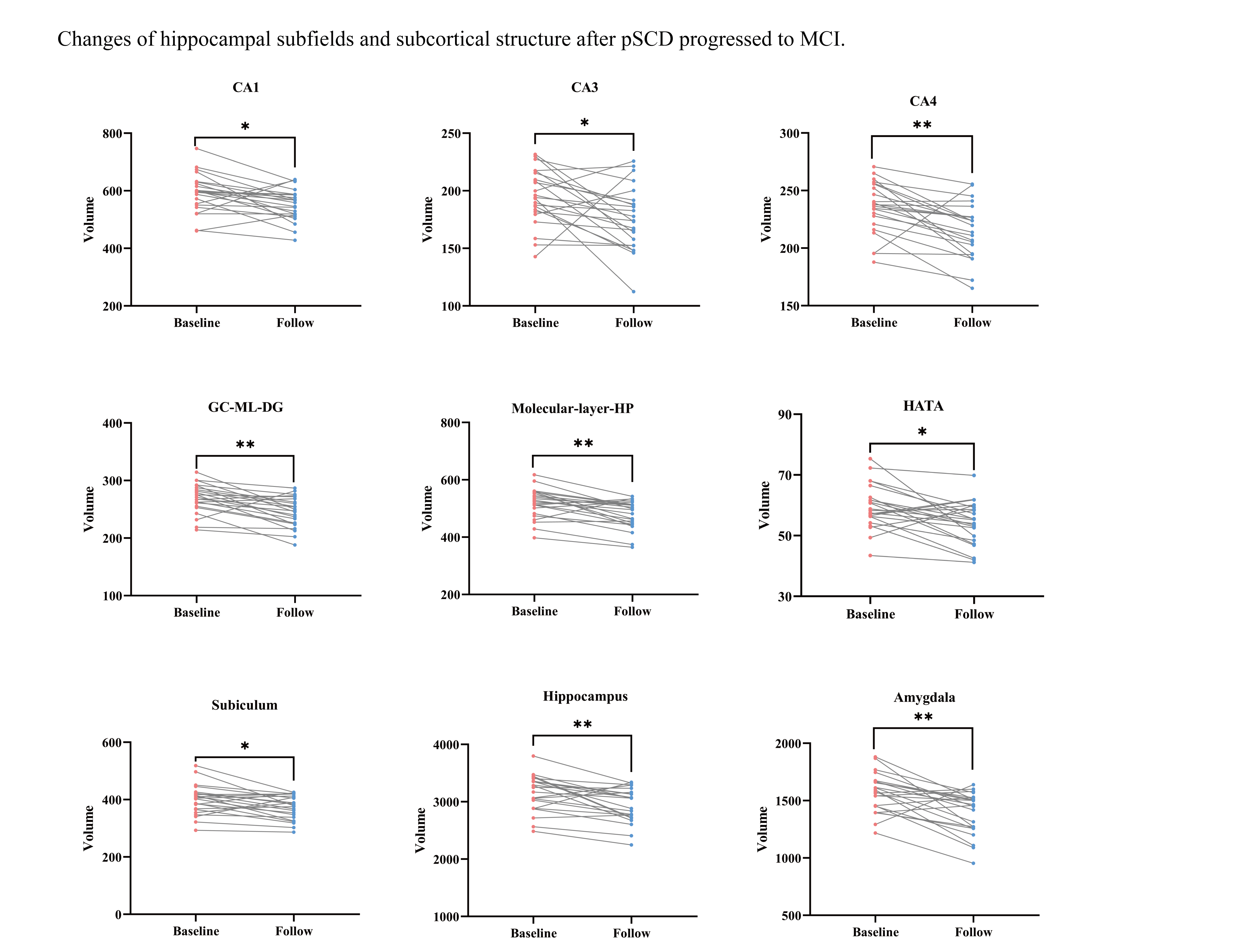

Supplement: Supplementary file 3 — Supplementary Material. [file BRB3-14-e3408-s002.tif]
